# Agreement between ChatGPT and human-derived multilevel meta-analyses: a reproducibility study across clinical evidence syntheses

**DOI:** 10.1186/s12874-026-02879-0

**Published:** 2026-06-05

**Authors:** Nikolai Ramadanov, Hannah Jaeger, Mikhail Salzmann, Robert Hable

**Affiliations:** 1 Center of Orthopaedics and Traumatology, Brandenburg Medical School, University Hospital Brandenburg, Brandenburg an der Havel, Germany; 2 Faculty of Health Science Brandenburg, Brandenburg Medical School Theodor Fontane, Brandenburg an der Havel, Germany; 3 Faculty of Applied Computer Science, Deggendorf Institute of Technology, Deggendorf, Germany

**Keywords:** ChatGPT, large language models, multilevel meta-analysis, reproducibility, evidence synthesis, digital health, research methodology

## Abstract

**Background:**

Large language models are increasingly integrated into biomedical research workflows, yet their ability to reliably reproduce complex statistical analyses remains insufficiently evaluated. While prior studies suggest that ChatGPT can replicate conventional meta-analyses, its performance in recalculating statistically demanding multilevel meta-analyses is unknown.

**Methods:**

We conducted a systematic review to identify published multilevel meta-analyses (i.e., models accounting for dependency among multiple effect sizes within studies) of hip-related clinical studies that provided extraction tables enabling independent recalculation. For each outcome, pooled estimates and heterogeneity parameters were recomputed using ChatGPT under predefined analytical assumptions and compared with statistician-derived results. Agreement was assessed using absolute and relative differences and Bland–Altman analyses for pooled effects, and qualitative comparison of heterogeneity measures (I², τ²) based on absolute differences.

**Results:**

Seven studies, each reporting a multilevel meta-analysis, comprising more than 40 pooled effect estimates were included. ChatGPT-derived pooled estimates showed close numerical agreement with human-derived results, with small median absolute differences for continuous (2.25) and binary outcomes (0.005) and minimal overall bias. Heterogeneity estimates demonstrated similar concordance, with predominantly small differences that did not alter qualitative interpretation. Larger deviations were limited to a small number of outcomes with extreme heterogeneity or inconsistent input structures.

**Conclusion:**

ChatGPT can reproducibly approximate the results of multilevel meta-analyses under predefined analytical conditions. These findings support the potential role of large language models as adjunct tools for reproducibility assessment and methodological validation in evidence synthesis, while emphasizing the continued need for expert oversight.

**Supplementary Information:**

The online version contains supplementary material available at https://doi.org/10.1186/s12874-026-02879-0.

## Introduction

Large language models (LLMs), such as ChatGPT, have rapidly entered biomedical research workflows and are increasingly being used beyond text generation tasks [1]. In the medical literature, ChatGPT has been evaluated for clinical question answering, patient education, scientific writing assistance, and methodological support. More recently, interest has shifted toward its potential role in quantitative research, including data extraction and statistical recalculation. Several studies have demonstrated that ChatGPT can reproduce results of conventional meta-analyses when provided with structured summary tables, suggesting that LLMs may assist in validating or reproducing established evidence synthesis methods [2, 3]. However, these investigations have largely been limited to standard fixed-effect or random-effects models.

Recent validation studies have demonstrated that large language models can approximate conventional frequentist meta-analytic calculations with a level of agreement comparable to established statistical software, particularly under predefined analytical conditions [4]. In parallel, other investigations have evaluated the performance of LLMs across different steps of the systematic review process, including study selection, data extraction, and statistical pooling, showing generally promising but variable results depending on task complexity [2]. A recent systematic review further highlights that, while LLMs perform well in structured and repetitive tasks, their reliability decreases in more complex analytical settings and requires careful human oversight [5].

Multilevel meta-analysis represents a methodological extension of conventional random-effects meta-analysis and is increasingly applied in clinical research fields characterized by complex data structures [6]. By explicitly modelling dependency among effect sizes - such as multiple outcomes, time points, or comparisons derived from the same study - multilevel meta-analyses allow more efficient use of available data while avoiding unit-of-analysis errors. At the same time, these models require iterative estimation procedures, additional variance components, and multiple analyst-defined assumptions, including correlation structures and inference methods. As a consequence, multilevel meta-analyses are statistically more complex and potentially more sensitive to analytical choices than conventional approaches.

Despite their growing popularity, the reproducibility of multilevel meta-analyses remains insufficiently examined. Previous methodological work has highlighted that many meta-analyses incompletely report key analytical decisions, which may limit the ability of independent researchers to reproduce published results even when extraction tables are available [7]. This concern is particularly relevant for multilevel meta-analyses, where small differences in model specification or estimation procedures may lead to non-trivial deviations in pooled estimates and heterogeneity parameters. To date, no study has systematically assessed whether the results of published multilevel meta-analyses can be recomputed using automated or semi-automated approaches based solely on reported summary data. Recent meta-research has shown that meta-analytic results are generally reproducible when original data files or analytic code are available, although practical limitations such as incomplete reporting and limited data accessibility remain common challenges [8, 9]. However, these studies primarily assess reproducibility within the same analytical framework, whereas the question of whether results can be independently approximated based solely on reported summary data remains insufficiently explored.

In parallel, there is currently no comparative literature evaluating the performance of ChatGPT in recalculating results from multilevel meta-analyses. While LLM-assisted recalculation has been explored for simpler meta-analytic models, it remains unknown whether ChatGPT can approximate the results of more advanced multilevel analyses when constrained to the same extraction tables used in the original publications. Furthermore, this question has not been investigated within a clearly defined clinical domain, such as hip surgery, where multilevel meta-analyses are increasingly employed to synthesize heterogeneous outcome data.

To our knowledge, this is the first study to systematically evaluate whether large language models can reproduce results of multilevel meta-analyses, thereby extending previous work that has been limited to conventional meta-analytic models or individual steps of the systematic review process.

Therefore, the aim of this study is twofold: first, to systematically review the literature for multilevel meta-analyses addressing hip-related conditions and surgical interventions; and second, to evaluate whether ChatGPT can recompute pooled estimates, confidence intervals, and heterogeneity parameters directly from the published extraction tables of these studies. By comparing ChatGPT-generated recalculations with the originally reported results using predefined tolerance thresholds, this study seeks to assess the reproducibility of multilevel meta-analyses and to determine the extent to which ChatGPT can replicate advanced meta-analytic computations based on reported summary data alone.

## Methods

### Study design and registration

This study was conducted as a systematic review of the literature followed by a reproducibility analysis of published multilevel meta-analyses. The protocol was developed in accordance with the PRISMA-P guidelines [10] and was registered in PROSPERO on 01 January 2026 (registration number: CRD420261276943). Reporting of this review followed the Preferred Reporting Items for Systematic Reviews and Meta-Analyses (PRISMA) 2020 guidelines [11]. The completed PRISMA 2020 checklist is provided in the Supplementary Appendix (Supplementary Table 1).

### Eligibility criteria

Studies were eligible if they meet the following criteria: (1) application of a multilevel meta-analytic model [6, 12] as the primary analytical approach; (2) focus on hip-related conditions or hip surgical interventions, including but not limited to femoroacetabular impingement syndrome (FAIS), developmental dysplasia of the hip (DDH), periacetabular osteotomy (PAO), hip arthroscopy (HAS), or total hip arthroplasty (THA); (3) availability of structured extraction tables or sufficient summary data to allow independent recalculation of pooled estimates, defined as reporting of study-level effect size inputs and corresponding measures of variability (e.g., means, standard deviations, and sample sizes for continuous outcomes, or event counts and total sample sizes for binary outcomes).

### Information sources and search strategy

A systematic literature search was performed in PubMed/MEDLINE, Embase, and Scopus from database inception to the date of the final search. Search terms were combined hip-related clinical concepts with methodological terms related to multilevel meta-analysis. The full search term was as follows: (((hip) OR (dysplasia) OR (developmental dysplasia) OR (DDH) OR (BDDH) OR (borderline developmental dysplasia) OR (femoroacetabular impingement) OR (FAI) OR (FAIS) OR (periacetabular osteotomy) OR (PAO) OR (total hip arthroplasty) OR (THA) OR (hip replacement) OR (hip arthroscopy)) AND ((multilevel meta-analysis) OR (multi-level meta-analysis))). The search strategy was adapted as appropriate for each database (Supplementary Table 2).

### Study selection

Titles and abstracts were screened independently by two reviewers (NR and HJ). Full-text articles were retrieved for all potentially eligible studies and were assessed independently by two reviewers (NR and HJ) against the predefined inclusion criteria. Disagreements were resolved by consensus or consultation with a third reviewer (RH).

### Data extraction

For each included multilevel meta-analysis, data were extracted using a standardized extraction form. Extracted information included the clinical topic, study design, number of included primary studies, number of effect sizes, reported pooled estimates, corresponding confidence intervals, heterogeneity measures, and all available extraction tables used in the original analyses. These extraction tables refer to structured datasets reporting study-level summary data used for meta-analytic pooling, typically including study identifiers, sample sizes, outcome measures (e.g., means and standard deviations), or event counts for binary outcomes. Data extraction was performed independently by two reviewers (NR and HJ).

### Methodological quality assessment

The methodological quality of the included multilevel meta-analyses was assessed using the AMSTAR 2 tool [13]. Quality assessments was performed independently by two reviewers (NR and HJ), and overall confidence ratings was assigned according to established AMSTAR 2 guidance.

### ChatGPT-based recalculation procedure

Using the raw data extraction tables of the included meta-analyses, ChatGPT (OpenAI, GPT-5) was instructed via the web interface (chat.openai.com) to recompute pooled effect estimates directly from the reported summary data under predefined and transparent model assumptions. Each row of the input table represented a single effect estimate, while individual studies could contribute one or multiple rows (e.g., different surgical strategies or outcomes), resulting in a hierarchical data structure with effect estimates nested within studies. ChatGPT was not provided with the originally published pooled results during the recalculation process.

For continuous outcomes, the input table included the following columns: study identifier, number of operated hips, surgical strategy (OP1–OP4), mean value, and standard deviation. For binary outcomes, the table included: study identifier, number of operated hips (total), surgical strategy (OP1–OP4), and number of events. Reanalyses were conducted within a frequentist multilevel random-effects meta-analytic framework, modelling study as a random effect to account for within-study clustering of multiple effect estimates. The predefined analytical assumptions included the use of a frequentist multilevel random-effects model, estimation of between-study variance (τ²) using the Sidik–Jonkman method, and statistical inference based on the Hartung–Knapp adjustment. Surgical strategy was included as a categorical study-level moderator, and differences between strategies were assessed using an omnibus test of moderators (QM). Reported outputs comprised pooled means (continuous outcomes) or pooled proportions (binary outcomes) with 95% confidence intervals, heterogeneity statistics (I², τ², and Cochran’s Q p-value), and moderator p-values. The full prompt used for recalculation is provided in Supplementary Appendix (Supplementary Table 3). A zero-shot prompting approach was used, meaning that no examples or prior guidance were provided to the model beyond the structured input data and predefined analytical instructions.

### Agreement analysis

Agreement between pooled results obtained by ChatGPT and those derived by an experienced statistician was assessed for both effect estimates and heterogeneity measures. The statistician was a member of the investigator team with experience in multilevel meta-analysis and was not involved in the original studies. For each analysis, the statistician used the same extraction tables and predefined analytical assumptions as those provided to ChatGPT and was not given access to the originally published pooled results during the recalculation process. For effect estimates (pooled means for continuous outcomes and pooled proportions for binary outcomes), agreement was evaluated using Bland–Altman analysis [14], reporting mean differences (bias) and 95% limits of agreement. In addition, absolute and relative differences between estimates were calculated descriptively.

Heterogeneity measures (I² and τ²) were compared between approaches based on absolute differences. Formal agreement testing was not applied to heterogeneity statistics, as these are derived measures sensitive to minor numerical and implementation differences. Consistency of heterogeneity estimates was interpreted qualitatively based on the magnitude and direction of observed differences.

## Results

### Systematic literature search

The literature search across PubMed, Embase, and Scopus yielded a total of 104 records (PubMed: 23; Embase: 45; Scopus: 36). After removal of 54 duplicate records, 50 unique records remained for title and abstract screening. Of these, 43 records were excluded, leaving 7 meta-analyses for full-text assessment [15–21]. No additional records were excluded following full-text review. Consequently, 7 meta-analyses were included in the final qualitative synthesis [15–21].

### Methodological quality assessment (AMSTAR-2)

The methodological quality of the seven included multilevel meta-analyses [15–21] was assessed using AMSTAR-2 (Tables 1 and 2). Overall confidence was rated as high for two meta-analyses [15, 18] and moderate for five meta-analyses [16, 17, 19–21]. No meta-analysis was rated as low or critically low quality.

**Table 1:** Methodological quality assessment of included multilevel meta-analyses using AMSTAR-2. Item-level ratings across all 16 AMSTAR-2 domains and overall confidence judgments (high or moderate) for each included study

AMSTAR-2 Domain	Conservative treatment versus hip arthroscopy in patients with femoroacetabular impingement (Bone & Joint Open 2025) [15]	Multilevel Meta-Analysis of Treatment Options for Iliopsoas Impingement Syndrome After THA (Orthopaedic Surgery 2025) [16]	Periacetabular Osteotomy vs. Hip Arthroscopy for BDDH (J Exp Orthop 2025) [17]	Postless versus traditional hip arthroscopy (KSSTA 2025) [18]	Capsular management (short-term) FAIS meta-analysis (KSSTA 2026) [19]	Pelvic tilt after PAO – single-arm multilevel meta-analysis (J Exp Orthop 2025) [20]	Capsular management (mid- to long-term) FAIS meta-analysis (KSSTA 2025) [21]
Item 1: Research questions and inclusion criteria include PICO	Yes – PICO clearly defined	Yes – PICO clearly defined	Yes – PICO clearly defined	Yes – PICO clearly defined	Yes – PICO clearly defined	Yes – population, intervention (PAO), outcome (pelvic tilt) clearly defined	Yes – population, interventions (CP/CR/CU), comparators and outcomes clearly defined
Item 2: Protocol registered before commencement	Yes – PROSPERO registration (CRD42024551224)	Yes – PROSPERO registered (CRD42024606752)	Yes – PROSPERO registered (CRD42025648749)	Yes – PROSPERO registered (CRD420251068473)	Yes – PROSPERO registered (CRD420251050197)	Yes – PROSPERO registered (CRD420251051267)	Yes – PROSPERO registered (CRD420251050197)
Item 3: Explanation for study design selection	Yes – RCT-only design justified	Yes – inclusion of prospective and retrospective studies justified	Yes – inclusion of RCTs and non-RCTs justified	Yes – inclusion of prospective and retrospective studies justified	Yes – RCTs and non-RCTs justified	Yes – inclusion of prospective and retrospective cohorts justified	Yes – RCTs and observational studies justified
Item 4: Comprehensive literature search strategy	Yes – multiple databases, no restrictions	Yes – PubMed, CENTRAL, Embase, Epistemonikos searched	Yes – PubMed, Embase, Epistemonikos; no restrictions	Yes – PubMed, Embase, Epistemonikos, CENTRAL searched	Yes – PubMed, Embase, CENTRAL, Epistemonikos	Yes – PubMed, Embase, Epistemonikos; no language restrictions	Yes – PubMed, Embase, CENTRAL, Epistemonikos; no language restrictions
Item 5: Study selection performed in duplicate	Yes – two independent reviewers	Yes – two independent reviewers, kappa reported	Yes – two independent reviewers, kappa reported	Yes – two independent reviewers, kappa reported	Yes – two independent reviewers, kappa reported	Yes – two independent reviewers, kappa reported	Yes – two independent reviewers, kappa reported
Item 6: Data extraction performed in duplicate	Yes – explicitly reported	Yes – explicitly reported	Yes – explicitly reported	Yes – explicitly reported	Yes – explicitly reported	Yes – explicitly reported	Yes – explicitly reported
Item 7: List of excluded studies with justification	Yes – PRISMA flow and reasons provided	Yes – PRISMA flow diagram with reasons	Yes – PRISMA flow with reasons	Yes – PRISMA flow diagram with reasons	Yes – PRISMA flow with reasons	Yes – PRISMA flow diagram with reasons	Yes – detailed PRISMA flow with reasons
Item 8: Description of included studies	Yes – detailed tables and characteristics	Yes – detailed characteristics table	Yes – detailed characteristics table	Yes – detailed study and patient characteristics tables	Yes – detailed tables	Yes – detailed characteristics tables	Yes – comprehensive tables of study and patient characteristics
Item 9: Risk of bias assessment of individual studies	Yes – Cochrane RoB 2 tool	Yes – ROBINS-I applied	Yes – ROBINS-I applied	Yes – ROBINS-I applied	Yes – RoB 2 and ROBINS-I	Yes – ROBINS-I applied	Yes – RoB 2 for RCTs and ROBINS-I for non-RCTs
Item 10: Reporting of funding sources of included studies	No – not systematically reported	No – funding of primary studies not systematically reported	No – funding of primary studies not systematically reported	No – funding of primary studies not systematically reported	No – not systematically reported	No – funding of primary studies not systematically reported	No – funding of primary studies not systematically reported
Item 11: Appropriate meta-analytical methods	Yes – multilevel random-effects, REML, Hartung–Knapp	Yes – multilevel random-effects, REML, Hartung–Knapp	Yes – multilevel random-effects, HKSJ estimator	Yes – multilevel random-effects, REML, Hartung–Knapp	Yes – multilevel random-effects, REML, Hartung–Knapp	Yes – single-arm multilevel random-effects, REML, Hartung–Knapp	Yes – multilevel random-effects, REML, Hartung–Knapp
Item 12: Assessment of impact of risk of bias on results	Yes – sensitivity analysis with low-RoB studies	Yes – GRADE and sensitivity considerations	Partial Yes – discussed, limited stratified analysis	Yes – sensitivity considerations and GRADE assessment	Partial Yes – discussed, limited stratification	Partial Yes – RoB described, limited stratified analyses	Partial Yes – RoB described, no formal RoB-stratified pooling
Item 13: Risk of bias considered in interpretation	Yes – discussed in Results and Discussion	Yes – discussed in Results and Discussion	Yes – explicitly addressed in discussion	Yes – explicitly addressed in Discussion	Yes – explicitly discussed	Yes – explicitly discussed in Results and Discussion	Yes – explicitly discussed
Item 14: Explanation and discussion of heterogeneity	Yes – I², τ², and interpretation provided	Yes – I², τ², meta-regression performed	Yes – I², τ², sensitivity and subgroup analyses	Yes – I², τ² and clinical interpretation provided	Yes – I², τ², subgroup and sensitivity analyses	Yes – I², τ², subgroup analyses, meta-regression	Yes – I², τ², subgroup analyses and clinical interpretation
Item 15: Investigation of publication bias	Yes – funnel plots for key outcomes	No – no formal publication bias analysis reported	Yes – funnel plots and Egger/Begg tests	Yes – funnel plots and asymmetry discussed	Yes – funnel plots and Begg test	Yes – funnel plots and Begg test reported	Yes – funnel plots visually assessed
Item 16: Declaration of conflicts of interest	Yes – conflicts of interest reported	Yes – conflicts of interest reported	Yes – conflicts of interest reported	Yes – conflicts of interest reported	Yes – conflicts of interest reported	Yes – conflicts of interest stated	Yes – conflicts of interest reported
Overall	High	Moderate	Moderate	High	Moderate	Moderate	Moderate

**Table 2:** Summary of critical AMSTAR-2 domains and overall confidence ratings. Selected key methodological items (protocol registration, search strategy, study selection, risk of bias assessment, appropriate meta-analytic methods, consideration of bias in interpretation, and publication bias assessment) and final overall quality judgments for each included study.

AMSTAR-2 Domain	Conservative treatment versus hip arthroscopy in patients with femoroacetabular impingement (Bone & Joint Open 2025) [15]	Multilevel Meta-Analysis of Treatment Options for Iliopsoas Impingement Syndrome After THA (Orthopaedic Surgery 2025) [16]	Periacetabular Osteotomy vs. Hip Arthroscopy for BDDH (J Exp Orthop 2025) [17]	Postless versus traditional hip arthroscopy (KSSTA 2025) [18]	Capsular management (short-term) FAIS meta-analysis (KSSTA 2026) [19]	Pelvic tilt after PAO – single-arm multilevel meta-analysis (J Exp Orthop 2025) [20]	Capsular management (mid- to long-term) FAIS meta-analysis (KSSTA 2025) [21]
Item 2	Yes	Yes	Yes	Yes	Yes	Yes	Yes
Item 4	Yes	Yes	Yes	Yes	Yes	Yes	Yes
Item 7	Yes	Yes	Yes	Yes	Yes	Yes	Yes
Item 9	Yes	Yes	Yes	Yes	Yes	Yes	Yes
Item 11	Yes	Yes	Yes	Yes	Yes	Yes	Yes
Item 13	Yes	Yes	Yes	Yes	Yes	Yes	Yes
Item 15	Yes	No	Yes	Yes	Yes	Yes	Yes
Overall	High	Moderate	Moderate	High	Moderate	Moderate	Moderate

All included meta-analyses [15–21] fulfilled the majority of AMSTAR-2 domains, including a clearly defined PICO framework (Item 1), prospective protocol registration (Item 2), justification of study design selection (Item 3), comprehensive literature search strategies (Item 4), duplicate study selection and data extraction (Items 5–6), provision of excluded studies with justification (Item 7), detailed description of included studies (Item 8), appropriate risk-of-bias assessment (Item 9), and use of suitable multilevel meta-analytic methods (Item 11).

The most frequent methodological limitation was the lack of systematic reporting of funding sources of the included primary studies (Item 10), which was not fulfilled in any meta-analysis [15–21]. Assessment of the impact of risk of bias on the results (Item 12) was partially [17, 19–21] or completely [15, 16, 18] fulfilled in several meta-analyses, whereas consideration of risk of bias in the interpretation of findings (Item 13) and exploration of heterogeneity (Item 14) were consistently [15–21] addressed. Publication bias (Item 15) was assessed in most meta-analyses [15, 18–21], and conflicts of interest (Item 16) were reported in all [15–21] studies.

### Comparison Chat GPT vs. human in multilevel meta-analyses

Across all outcomes, ChatGPT-derived pooled estimates showed close numerical agreement with statistician-derived results (Table 3). For continuous outcomes, the median absolute difference was 2.25, corresponding to a median relative difference of 8.62%, while binary outcomes demonstrated very small discrepancies (median absolute difference 0.005) (Table 4). Bland–Altman analyses indicated minimal overall bias, suggesting no systematic over- or underestimation by ChatGPT. Larger deviations were limited to a small number of outlying outcomes, whereas the majority of effect estimates differed only marginally (Table 4). Overall, these findings indicate generally good agreement between approaches, although notable variability was observed for continuous outcomes, particularly in comparisons with large dispersion or extreme heterogeneity. In contrast, binary outcomes demonstrated consistently small absolute differences and closer agreement. Detailed results are presented in Tables 3 and 4.

**Table 3:** Comparison of pooled effect estimates derived from ChatGPT and statistician-based analyses. The test for subgroup differences (QM) reflects differences between surgical strategies (OP1–OP4) within the multilevel meta-analytic model and does not represent a comparison between ChatGPT and statistician-derived estimates.

Study	Outcome	Mean (95%-CIs)	Heterogeneity I²	Heterogeneity τ²	Heterogeneity *p*	Test for subgroup differences
Human [17]	mHHS	84.47 [82.34–86.60]	87%	30.58	< 0.001	0.46
Chat GPT		84.15 95% CI: 83.38–84.92	86.39%	18.85	n/a	0.998
Human [17]	iHOT-12	73.74 [68.61–78.87]	95%	44.09	< 0.01	0.98
Chat GPT		74.44 95% CI: 72.68–76.20	91.43%	45.88	< 0.001	0.674
Human [17]	Diff. mHHS	23.76 [21.23–26.29]	86%	41.12	< 0.01	0.54
Chat GPT		24.00 95% CI: 23.02–24.97	85.50%	41.26	< 0.001	0.442
Human [17]	Diff. iHOT-12	34.97 [27.46–42.49]	91%	69.79	< 0.01	0.30
Chat GPT		33.35 95% CI: 31.35–35.35	91.27%	79.46	< 0.001	0.547
Human [17]	Functional MCID	9.92 [9.46–10.37]	97%	1.72	< 0.01	0.51
Chat GPT		10.10 95% CI: 10.01–10.19	95.17%	0.91	< 0.001	0.937
Human [19]	mHHS change	22.27 [19.61–24.93]	96%	48.41	< 0.01	0.01
Chat GPT		20.35 95% CI: 18.70–21.99	95.88%	81.20	< 0.001	0.376
Human [19]	Functional MCID	8.55 [7.52–9.58]	100%	4.78	0.0	0.04
Chat GPT		8.88 95% CI: 8.76–9.00	99.34%	1.28	< 0.001	0.0064
Human [18]	VAS	3.04 [0.41–5.67]	95%	1.61	< 0.01	0.58
Chat GPT		3.48 95% CI: 3.26–3.70	94.93%	1.0013	< 0.001	0.512
Human [18]	Traction time	55.9 [39.04–72.76]	99%	195.36	< 0.01	< 0.01
Chat GPT		53.30 95% CI: 52.22–54.38	98.54%	121.18	< 0.001	0.154
Human [20]	PT preoperative	8.13 [5.40–10.86]	98%	23.48	< 0.01	N/A
Chat GPT		10.38 95% CI: 9.48–11.27	95.01%	12.76	< 0.001	N/A
Human [20]	PT postperative	6.81 [3.38–10.23]	93%	15.15	< 0.01	N/A
Chat GPT		10.38 95% CI: 9.48–11.27	95.01%	12.76	< 0.001	N/A
Human [20]	PT change	0.81 [-0.64–2.27]	35%	1.39	0.13	N/A
Chat GPT		10.38 95% CI: 9.48–11.27	95.01%	12.76	< 0.001	N/A
Human [15]	HHS ≤ 12 months post-intervention	85.30 [77.7–92.91]	93%	38.92	< 0.01	0.02
Chat GPT		82.34 95% CI: 81.43–83.26	93.36%	13.40	< 0.001	0.849
Human [15]	iHOT ≤ 24 months post-intervention	62.13 [54.89–69.37]	91%	143.74	< 0.01	0.04
Chat GPT		58.71 95% CI: 56.98–60.43	91.13%	141.86	< 0.001	0.252
Human [15]	Functional MCID	7.42 [6.23–8.61]	99%	6.26	0.0	0.13
Chat GPT		6.95 95% CI: 6.71–7.20	99.16%	5.92	< 0.001	0.114
Human [15]	Pain MCID	1.36 [1.07–1.65]	87%	0.18	< 0.01	0.20
Chat GPT		1.41 95% CI: 1.32–1.50	87.10%	0.14	< 0.001	0.231
Human [16]	HHS post	82.24 [75.4–89.08]	95%	79.02	< 0.01	0.20
Chat GPT		7.67 95% CI: 5.65–9.69	61.44%	6.36	0.0038	0.0318
Human [16]	HHS change	25.75 [16.83–34.67]	94%	117.65	< 0.01	0.22
Chat GPT		7.00 95% CI: 4.98–9.02	56.85%	4.94	0.0133	0.0508
Human [16]	Functional MCID	9.04 [7.59–10.5]	100%	7.24	0.0	0.42
Chat GPT		13.00 95% CI: 11.01–14.99	79.60%	22.67	< 0.001	0.0021
Human [21]	mHHS 2.5–10 years postoperatively	81.14 [77.72–84.56]	87%	15.49	< 0.01	0.67
Chat GPT		6.33 95% CI: 4.30–8.36	51.12%	3.66	0.0374	0.0507
Human [21]	HOS ADL 2.5–10 years postoperatively	87.94 [84.79–91.09]	77%	10.9	< 0.01	0.90
Chat GPT		6.33 95% CI: 4.30–8.36	51.12%	3.66	0.0374	0.0737
Human [21]	HOS SSS 2.5–10 years postoperatively	78.59 [72.69–84.48]	86%	29.68	< 0.01	0.84
Chat GPT		6.33 95% CI: 4.30–8.36	51.12%	3.66	0.0374	0.0737
Human [21]	VAS 2.5–10 years postoperatively	2.42 [2.08–2.77]	52%	0.05	0.06	0.51
Chat GPT		4.33 95% CI: 2.27–6.40	20.71%	0.71	0.278	0.126
Human [17]	Reoperation	0.09 [0.06–0.13]	74%	0.93	< 0.01	0.68
Chat GPT		0.093 95% CI: 0.066–0.130	74.3%	0.73	< 0.001	0.566
Human [17]	Complications	0.04 [0.02–0.08]	80%	0.84	< 0.01	0.07
Chat GPT		0.033 95% CI: 0.012–0.084	80.1%	2.42	< 0.001	0.210
Human [18]	Nerve injury and numbness	0.03 [0.00–0.27]	91%	7.24	< 0.01	0.02
Chat GPT		0.096 95% CI: 0.041–0.209	91.12%	1.47	< 0.001	0.043
Human [18]	Sexual and urinary dysfunction	0.02 [0.00–0.11]	57%	1.42	0.04	0.34
Chat GPT		0.019 95% CI: 0.006–0.064	56.6%	1.28	0.042	0.653

### Agreement of pooled effect estimates

Across all continuous outcome parameters included in the agreement table, ChatGPT-derived pooled estimates showed close numerical concordance with statistician-derived results for the majority of outcomes. The median absolute difference between estimates was 2.25, with a median relative difference of 8.62%. Bland–Altman analysis demonstrated an overall mean difference of − 13.04 with a standard deviation of 40.52, reflecting substantial variability driven by a small number of outlying comparisons (Tables 4 and 5).

**Table 4:** Summary of agreement metrics between ChatGPT- and statistician-derived analyses. Median absolute differences, ranges, relative differences, and Bland–Altman bias estimates for pooled effects, statistical heterogeneity (I²), and between-study variance (τ²) across continuous and binary outcomes

Continuous Outcome	Human estimate	ChatGPT estimate	Absolute difference	Relative difference (%)
mHHS [17]	84,47	84,15	0,32	0,38
HHS [15]	85,30	82,34	2,96	3,47
HHS post [16]	82,24	7,67	74,57	90,66
mHHS [21]	81,14	6,33	74,81	92,19
mHHS change [17]	23,76	24,00	0,24	1,01
mHHS change [19]	22,27	20,35	1,92	8,62
HHS change [16]	25,75	7,00	18,75	72,82
iHOT-12 [17]	73,74	74,44	0,70	0,95
iHOT [15]	62,13	58,71	3,42	5,50
iHOT-12 change [17]	34,97	33,35	1,62	4,63
HOS ADL [21]	87,94	6,33	81,61	92,80
HOS SSS [21]	78,59	6,33	72,26	91,95
Functional MCID [17]	9,92	10,10	0,18	1,81
Functional MCID [19]	8,55	8,88	0,33	3,86
Functional MCID [16]	9,04	13,00	3,96	43,81
Functional MCID [15]	7,42	6,95	0,47	6,33
VAS [19]	3,04	3,48	0,44	14,47
VAS [21]	2,42	4,33	1,91	78,93
Pain MCID [15]	1,36	1,41	0,05	3,68
Traction time [18]	55,90	53,30	2,60	4,65
PT preoperative [20]	8,13	10,38	2,25	27,68
PT postoperative [20]	6,81	10,38	3,57	52,42
PT change [20]	0,81	10,38	9,57	1181,48

Binary Outcome	Human estimate	ChatGPT estimate	Absolute difference	Relative difference (%)
Reoperation [17]	0,09	0,093	0,003	3,33
Complications [17]	0,04	0,033	0,007	17,50
Nerve injury & numbness [18]	0,03	0,096	0,066	220,00
Sexual & urinary dysfunction [18]	0,02	0,019	0,001	5,00

Across binary outcome parameters, ChatGPT-derived pooled proportions showed close agreement with statistician-derived estimates. The median absolute difference between proportions was 0.005 (range 0.001–0.066), corresponding to a median relative difference of 11.25%. Bland–Altman analysis demonstrated a small positive bias of 0.015 with a standard deviation of differences of 0.034, indicating no systematic deviation between approaches (Tables 4 and 5).

**Table 5:** Absolute and relative differences between ChatGPT- and statistician-derived pooled effect estimates. For each continuous and binary outcome, human and ChatGPT estimates are shown alongside absolute and percentage differences to quantify agreement between approaches

Continuous outcome	I² Human (%)	I² ChatGPT (%)	ΔI²
mHHS [17]	87.00	86.39	−0.61
HHS [15]	93.00	93.36	+ 0.36
HHS post [16]	95.00	61.44	−33.56
mHHS [21]	87.00	51.12	−35.88
mHHS change [17]	86.00	85.50	−0.50
mHHS change [19]	96.00	95.88	−0.12
HHS change [16]	94.00	56.85	−37.15
iHOT-12 [17]	95.00	91.43	−3.57
iHOT [15]	91.00	91.13	+ 0.13
iHOT-12 change [17]	91.00	91.27	+ 0.27
HOS ADL [21]	77.00	51.12	−25.88
HOS SSS [21]	86.00	51.12	−34.88
Functional MCID [17]	97.00	95.17	−1.83
Functional MCID [19]	100.00	99.34	−0.66
Functional MCID [16]	100.00	79.60	−20.40
Functional MCID [15]	99.00	99.16	+ 0.16
VAS [18]	95.00	94.93	−0.07
VAS [21]	52.00	20.71	−31.29
Pain MCID [15]	87.00	87.10	+ 0.10
Traction time [18]	99.00	98.54	−0.46
PT preoperative [20]	98.00	95.01	−2.99
PT postoperative [20]	93.00	95.01	+ 2.01
PT change [20]	35.00	95.01	+ 60.01

Binary outcome	I² Human (%)	I² ChatGPT (%)	ΔI² (ChatGPT − Human)
Reoperation [17]	74.00	74.30	+ 0.30
Complications [17]	80.00	80.10	+ 0.10
Nerve injury & numbness [18]	91.00	91.12	+ 0.12
Sexual & urinary dysfunction [18]	57.00	56.60	−0.40

### Agreement of heterogeneity measures (I²)

Across all continuous outcome parameters, I² estimates derived by ChatGPT showed close numerical agreement with statistician-derived values. The median absolute difference in I² was 0.36, with a wide overall range (− 37.15 to + 60.01) driven by a small number of outcomes with extreme heterogeneity. For the majority of outcomes, differences in I² were minimal and did not result in any qualitative change in heterogeneity interpretation (Tables 4 and 6).

**Table 6:** Agreement of statistical heterogeneity (I²) between ChatGPT- and statistician-derived analyses. For each outcome, human and ChatGPT I² values and their differences (ΔI²) are presented to quantify concordance between approaches.

Continuous outcome	τ² Human	τ² ChatGPT	Δτ² (ChatGPT − Human)
mHHS [17]	40.56	18.85	−21.71
HHS [15]	38.92	13.40	−25.52
HHS post [16]	79.02	6.36	−72.66
mHHS [21]	15.49	3.66	−11.83
mHHS change [17]	41.12	41.26	+ 0.14
mHHS change [19]	48.41	81.20	+ 32.79
HHS change [16]	117.65	4.94	−112.71
iHOT-12 [17]	44.09	45.88	+ 1.79
iHOT [15]	143.74	141.86	−1.88
iHOT-12 change [17]	69.79	79.46	+ 9.67
HOS ADL [21]	10.90	3.66	−7.24
HOS SSS [21]	29.68	3.66	−26.02
Functional MCID [17]	1.72	0.91	−0.81
Functional MCID [19]	4.78	1.28	−3.50
Functional MCID [16]	7.24	22.67	+ 15.43
Functional MCID [15]	6.26	5.92	−0.34
VAS [18]	1.61	1.0013	−0.61
VAS [21]	0.05	0.71	+ 0.66
Pain MCID [15]	0.18	0.14	−0.04
Traction time [18]	195.36	121.18	−74.18
PT preoperative [20]	23.48	12.76	−10.72
PT postoperative [20]	15.15	12.76	−2.39
PT change [20]	1.39	12.76	+ 11.37

Binary outcome	τ² Human	τ² ChatGPT	Δτ² (ChatGPT − Human)
Reoperation [17]	0,93	0,73	−0,20
Complications [17]	0,84	2,42	+ 1,58
Nerve injury & numbness [18]	7,24	1,47	−5,77
Sexual & urinary dysfunction [18]	1,42	1,28	−0,14

Across binary outcome parameters, I² estimates derived by ChatGPT demonstrated very close numerical agreement with statistician-derived values. The median absolute difference in I² was 0.21, with a narrow overall range (− 0.40 to + 0.30). No systematic directional differences were observed, and heterogeneity interpretation remained unchanged across all binary outcomes (Tables 4 and 6).

### Agreement of between-study variance (τ²)

Across all continuous outcome parameters, between-study heterogeneity estimates (τ²) derived by ChatGPT showed close numerical agreement with statistician-derived values. The median absolute difference in τ² was 9.67, with a wide overall range (0.04–112.71) driven by a limited number of outcomes with very large heterogeneity estimates. For the majority of outcomes, absolute differences in τ² were small and did not result in any qualitative change in the interpretation of between-study heterogeneity (Tables 4 and 7).

Across binary outcome parameters, between-study heterogeneity estimates (τ²) derived by ChatGPT showed close numerical agreement with statistician-derived values. The median absolute difference in τ² was 0.89, with an overall range of 0.14–5.77 driven mainly by one outcome with higher between-study variance. For the remaining outcomes, absolute differences were small and did not affect the qualitative interpretation of heterogeneity (Tables 4 and 7).

**Table 7:** Agreement of between-study variance estimates (τ²) between ChatGPT- and statistician-derived analyses. For each outcome, human and ChatGPT τ² values and their differences (Δτ²) are presented to quantify concordance of between-study heterogeneity estimates

Effects	Median absolute difference Δ	Range	Median relative difference	Mean difference (bias, ChatGPT − Human)	SD of differences
Continuous	2.25	0.05 to 81.61	8.62	-13.04	40.52
Binary	0.005	0.001 to 0.066	11.25	0.015	0.034

I²	Median absolute difference Δ	Range	Median relative difference	Median ΔI² (ChatGPT − Human)	Direction of difference
Continuous	0.36	-37.15 to 60.01	N/A	-0.36	predominantly slightly lower I² values with ChatGPT
Binary	0.21	-0.40 to 0.30	N/A	0.11	no systematic deviation

τ²	Median absolute difference (Δτ²)	Range	Median relative difference	Median Δτ² (ChatGPT − Human)	Direction of difference
Continuous	9.67	0.04 to 112.71	N/A	-0.61	predominantly lower τ² values with ChatGPT
Binary	0.89	0.14 to 5.77	N/A	-0.17	predominantly lower τ² values with ChatGPT

## Discussion

The present study evaluated whether ChatGPT can recompute results of published multilevel meta-analyses when constrained to the same extraction tables and predefined analytical assumptions as those used by an experienced statistician. Across a set of multilevel meta-analyses in hip-related surgery, ChatGPT-derived recalculations demonstrated a high degree of numerical concordance with the originally reported results for pooled effect estimates as well as for heterogeneity measures. Importantly, agreement was assessed using appropriate metrics tailored to the nature of the parameters under investigation, distinguishing between effect estimates and derived heterogeneity statistics.

For continuous outcomes, pooled effect estimates showed small absolute and relative differences in most cases, with median deviations well within ranges that are unlikely to be clinically or methodologically meaningful. Bland–Altman analysis revealed minimal systematic bias between ChatGPT-derived and statistician-derived results, indicating that ChatGPT did not consistently overestimate or underestimate pooled effects. Larger deviations were confined to a limited number of outcomes and were driven by clearly identifiable issues such as scale mismatches or the combination of final values and change scores. These discrepancies highlight that ChatGPT, like any analytical tool, is sensitive to the structure and internal consistency of the input data rather than indicating a fundamental limitation in its computational capability.

Binary outcomes demonstrated even closer agreement, with very small absolute differences between pooled proportions. Although relative differences appeared larger in some cases, this effect was attributable to low event rates and small absolute denominators rather than to meaningful discrepancies in the underlying estimates. From a methodological perspective, ChatGPT’s performance in recalculating binary outcomes was comparable to that of conventional statistical approaches, provided that event counts and denominators were clearly specified.

Heterogeneity measures required a more nuanced interpretation. Differences in I² values between ChatGPT-derived and statistician-derived analyses were generally small, with median absolute differences close to zero. Larger deviations were observed only for outcomes with extreme heterogeneity, where I² is known to be unstable and highly sensitive to minor numerical changes. Crucially, these numerical differences did not lead to any qualitative change in heterogeneity interpretation; outcomes classified as having high heterogeneity remained highly heterogeneous, and those with low or moderate heterogeneity retained their qualitative classification. This finding supports the view that ChatGPT can reproduce the overall heterogeneity signal of multilevel meta-analyses, even if exact percentage values differ slightly.

For τ², absolute differences were larger and more variable, particularly for outcomes with large between-study variance. This behavior is expected, as τ² is a scale-dependent parameter that can vary substantially with small changes in model specification, estimation procedure, or outcome scaling. Notably, ChatGPT tended to slightly underestimate the between-study variance τ² in many cases. However, as with I², these differences did not alter the substantive interpretation of heterogeneity and should be understood as numerical rather than conceptual discrepancies.

Taken together, these findings suggest that ChatGPT is capable of approximating the results of multilevel meta-analyses with a degree of accuracy that is comparable to that of human analysts, when provided with structured extraction tables and clearly defined analytical instructions. The observed deviations are consistent with known sensitivities of multilevel models to data structure and scaling and are not indicative of systematic computational errors. Importantly, the results emphasize that ChatGPT does not autonomously resolve ambiguities or inconsistencies in input data; careful preparation and validation of extraction tables remain essential. Crucially, the aim of this approach is not to replicate the original analysis using identical statistical software, but to assess whether published results can be independently approximated based solely on reported summary data and transparent analytical assumptions.

This study extends previous work on LLM-assisted meta-analytic recalculation by focusing specifically on multilevel models, which are statistically more complex and less frequently reproducible than conventional random-effects meta-analyses. By demonstrating that ChatGPT can recompute pooled estimates and heterogeneity parameters from published extraction tables, the present findings highlight a potential role for LLMs as supportive tools in reproducibility checks, sensitivity analyses, and educational contexts. However, these tools should be viewed as complements to, rather than replacements for, expert statistical oversight.

In contrast to prior validation studies that focused on conventional meta-analytic models, where LLMs showed acceptable agreement under controlled conditions (DOI: https://doi.org/10.1002/ksa.70379), the present study specifically evaluates performance in the more complex setting of multilevel meta-analysis. Given that previous work has identified increasing variability of LLM performance with task complexity across systematic review tasks (DOI: https://doi.org/10.1136/rapm-2024-106358; DOI: https://doi.org/10.1016/j.jclinepi.2026.112221), our findings provide important additional evidence by demonstrating that such models can approximate not only pooled effect estimates but also heterogeneity parameters within a multilevel framework.

### Limitations

Several limitations merit consideration. First, the analysis was restricted to hip-related multilevel meta-analyses, and generalizability to other clinical domains remains to be established. Second, ChatGPT was evaluated under highly controlled conditions with predefined prompts and explicit analytical assumptions; performance may differ in less structured settings. Third, although ChatGPT was not provided with the original pooled results, it cannot be fully excluded that elements of the included studies were part of its training data, which may have influenced recalculation performance and represents a potential source of bias. Finally, the study focused on numerical agreement rather than on the interpretative or decision-making aspects of meta-analysis, which remain inherently dependent on human judgment. In addition, the relatively small number of included multilevel meta-analyses may limit the precision of the observed differences and restrict the robustness of the findings.

### Future directions

Future research should evaluate the performance of large language models in multilevel meta-analyses across different clinical domains and with larger and more diverse datasets. In addition, further work is needed to assess performance under less controlled conditions and to explore the integration of LLMs into real-world evidence synthesis workflows. Finally, methodological studies should investigate the robustness of LLM-based recalculations under varying model specifications and analytical assumptions.

## Conclusion

In conclusion, ChatGPT can reproducibly approximate the results of published multilevel meta-analyses under predefined analytical conditions, although these findings should be interpreted with caution given the limited sample size and require confirmation in larger datasets. While it cannot substitute for methodological expertise or critical appraisal, it may serve as a valuable adjunct for reproducibility assessment and methodological validation in evidence synthesis.

## Supplementary Information

Supplementary Material 1. Supplementary Table 1: PRISMA 2020 checklist. Completed Preferred Reporting Items for Systematic Reviews and Meta-Analyses (PRISMA) 2020 checklist for the present study.

Supplementary Material 2. Supplementary Table 2: Full search strategy for PubMed/MEDLINE, Embase, and Scopus. The complete electronic search strategy, including all search terms and Boolean operators, used to identify eligible studies for this systematic review.

Supplementary Material 3. Supplementary Table 3: Full ChatGPT prompt for multilevel meta-analytic recalculation. The complete prompt, including input structure, model assumptions, and requested outputs, used to instruct ChatGPT to recompute pooled estimates and heterogeneity parameters from the extraction tables.

## Data Availability

No datasets were generated or analysed during the current study.

## Abbreviations

AMSTAR 2: A Measurement Tool to Assess Systematic Reviews 2
BDDH: Borderline Developmental Dysplasia of the Hip
CI: Confidence Interval
DDH: Developmental Dysplasia of the Hip
FAI: Femoroacetabular Impingement
FAIS: Femoroacetabular Impingement Syndrome
HAS: Hip Arthroscopy
I²: Inconsistency Index
LLM: Large Language Model
MCID: Minimal Clinically Important Difference
NR: Nikolai Ramadanov
PAO: Periacetabular Osteotomy
PICO: Population, Intervention, Comparator, Outcome
PRISMA: Preferred Reporting Items for Systematic Reviews and Meta–Analyses
PRISMA: P–Preferred Reporting Items for Systematic Review and Meta–Analysis Protocols
PROSPERO: International Prospective Register of Systematic Reviews
QM: Test of Moderators (Cochran’s Q for Model)
Q: Cochran’s Q Statistic
SD: Standard Deviation
THA: Total Hip Arthroplasty
τ²: Between–study Variance
