# Supplementary material for: Agreement between ChatGPT and human-derived multilevel meta-analyses: a reproducibility study across clinical evidence syntheses
Source: BMC Med Res Methodol. 2026 Jun 5;26:211. doi: 10.1186/s12874-026-02879-0 (PMC13621747; doi:10.1186/s12874-026-02879-0)
Supplement: Supplementary file 2 [file 12874_2026_2879_MOESM2_ESM.docx]

| Full electronic search strategies | |
| --- | --- |
| PubMed/MEDLINE | (((hip) OR (dysplasia) OR (developmental dysplasia) OR (DDH) OR (BDDH) OR (borderline developmental dysplasia) OR (femoroacetabular impingement) OR (FAI) OR (FAIS) OR (periacetabular osteotomy) OR (PAO) OR (total hip arthroplasty) OR (THA) OR (hip replacement) OR (hip arthroscopy)) AND ((multilevel meta-analysis) OR (multi-level meta-analysis))) |
| Embase | (('hip'/exp OR hip OR dysplasia OR 'developmental dysplasia' OR ddh OR bddh OR 'borderline developmental dysplasia' OR 'femoroacetabular impingement'/exp OR fai OR fais OR 'periacetabular osteotomy' OR pao OR 'total hip arthroplasty'/exp OR tha OR 'hip replacement' OR 'hip arthroscopy'/exp) AND ('multilevel meta analysis' OR 'multi level meta analysis')) |
| Scopus | (TITLE-ABS-KEY(hip OR dysplasia OR "developmental dysplasia" OR DDH OR BDDH OR "borderline developmental dysplasia" OR "femoroacetabular impingement" OR FAI OR FAIS OR "periacetabular osteotomy" OR PAO OR "total hip arthroplasty" OR THA OR "hip replacement" OR "hip arthroscopy") AND TITLE-ABS-KEY("multilevel meta-analysis" OR "multi-level meta-analysis")) |
