# Supplementary material for: Agreement between ChatGPT and human-derived multilevel meta-analyses: a reproducibility study across clinical evidence syntheses
Source: BMC Med Res Methodol. 2026 Jun 5;26:211. doi: 10.1186/s12874-026-02879-0 (PMC13621747; doi:10.1186/s12874-026-02879-0)
Supplement: Supplementary file 3 [file 12874_2026_2879_MOESM3_ESM.docx]

**ChatGPT, please perform a multilevel meta-analysis based on the attached table.**

**Data structure**

**The table contains one row per study and exactly one surgical strategy per study.
Columns are defined as follows:**

- **Study: study identifier**
- **n_hips: number of operated hips**
- **group: surgical procedure**
  - **1 = periacetabular osteotomy (PAO)**
  - **2 = hip arthroscopy (HAS)**
- **mHHS_mean: mean modified Harris Hip Score at final follow-up**
- **mHHS_SD: standard deviation of mHHS**

**All mHHS values represent final follow-up scores (not change scores).**

**Statistical methods**

- **Frequentist meta-analytic framework**
- **Random-effects multilevel (study-level) model**
- **Outcome: mHHS (continuous, mean values)**
- **Within-study variance calculated as SD² / n**
- **Between-study heterogeneity estimated as τ² (Sidik–Jonkman estimator)**
- **Surgical procedure (PAO vs HAS) included as a study-level moderator**
- **Inference using Hartung–Knapp adjustment**

**Please calculate and report only the following:**

- **Overall pooled mean mHHS (PAO + HAS) with 95% CI**
- **Heterogeneity I²**
- **Heterogeneity τ²**
- **Heterogeneity p-value**
- **Test for subgroup differences (PAO vs HAS, p-value)**
